# Normal Range of Thoracic Kyphosis in Male School Children

**DOI:** 10.1155/2014/159465

**Published:** 2014-04-06

**Authors:** MohammadBagher Shamsi, Korosh Veisi, Loghman Karimi, Javad Sarrafzadeh, Farid Najafi

**Affiliations:** ^1^Rehabilitation and Sport Medicine Department, School of Paramedicine, Kermanshah University of Medical Sciences, Kermanshah 6715847141, Iran; ^2^Physiotherapy Department, School of Rehabilitation Sciences, Iran University of Medical Sciences, Mohseni Square, Tehran 439115875, Iran; ^3^Department of Sport Management, Kurdestan Research and Science Branch, Islamic Azad University, Sanandaj 6616964353, Iran; ^4^Saqqez Education Office, Saqqez 6681763761, Iran; ^5^Kermanshah Health Research Center, School of Population Health, Kermanshah University of Medical Sciences, Kermanshah 6715847141, Iran

## Abstract

*Background*. Although there are frequent studies about normal range of thoracic kyphosis, there is still a controversy about the exact values of this curve. In nine reported studies on 10 to 20 years of age boys, the value ranged from 25.1° to 53.3°. *Objective for the Study*. The aim of the present research was investigation of normal ranges of thoracic kyphosis in school children in Kermanshah, western Iran. *Methods*. 582 male students aged 13 to 18 years old using cluster random sampling were recruited from schools in Kermanshah city, 97 students for each age. Thoracic curves were measured using the flexicurve method. *Results*. Mean thoracic kyphosis for whole population was 35.49° SD 7.83 and plus or minus two standard deviations ranged from 19.83° to 51.15°. It increased gradually from 13 to 16 and then there was a little decrease to 18 years. Mean values for each age (13–16) were 13 (34.41 SD 7.47°), 14 (34.86 SD 8.29°), 15 (35.79 SD 7.93°), 16 (36.49 SD 7.85°), 17 (35.84 SD 8.33°), and 18 (35.55 SD 7.07°). *Conclusions*. Our results are in agreement with previous reports and can be used as normal values for local and regional purposes.

## 1. Introduction

The thoracic angle is the primary curve of the vertebral column which is comprised of 12 vertebrae [1]. The thoracic kyphosis angle increases with age and the increase is greater in females than in males [2, 3]. Hyperkyphosis or increase in thoracic curve greater than normal range is one of prevalent spinal disorders. Biomechanical data suggest that an increase in the thoracic kyphosis may be associated with significantly higher spinal loads and trunk muscle force in upright stance and this might accelerate degenerative process which in turn leads to further spinal dysfunction and pain [4]. An increase in thoracic kyphosis has also been associated with diminished physical function [5], impairment of respiratory function [6, 7], an increase in cervical pain [8, 9], headaches [10], and shoulder discomforts such as subacromial pain syndrome [11, 12].

In spite of frequent studies on normal range of thoracic angle, there is a controversy about the magnitude of this curve. For example in Willner and Johnson [13] study the least pronounced kyphosis was seen at the age of 10–12 years and mean kyphosis angle in 8 and 16 years was 35° and 44°, respectively [13]. Propst Proctor and Bleck [14] in a study on children aged from 2 to 20 years old reported an angle of 27° (range of 21–33°) as normal. The magnitude of thoracic kyphosis in the study of Voutsinas and MacEwen [15] averaged 38.l° in white boys and 34.3° in black boys and 38.5° in white girls and 36.9° in black girls. In adults, kyphosis curve values varied according to different investigators, with the range of ~35°–37° [16, 17], but such studies were conducted in heterogeneous populations. Boseker et al. [18] using ± two standard deviations reported that normal kyphosis is ranged from 20° to 50°.

Such wide variations may be partly related to factors that affect spinal curve measurement such as difference in the instrument used and sample characteristic (age, sex,…), and limitations researchers have been faced with and partly related to factors that have been claimed to have influence on the curve such as age [19], sex [19], geographical region [17], and race [15].

Although there are quite different studies around the world on ranges of kyphosis, it seems that each region needs its specific values. The aim of this study was to define normal kyphosis range in 13–18-year-old male students living in Kermanshah, a city in the west of Iran.

## 2. Materials and Methods

The Ethical Board of Kermanshah University of Medical Sciences approved this study. For this purpose, 582 subjects were chosen across male students aged 13 to 18 years living in Kermanshah. Using random cluster sampling 6 guidance schools, 6 high schools were chosen. In each school, for every age group (13–18), at least 16 students were defined randomly. Exclusion criteria were having neuromuscular disorders, scoliosis, history of surgery, or any diseases on spinal column. In addition, if the students suffered from severe kyphosis, they were excluded.

**Figure 1:**
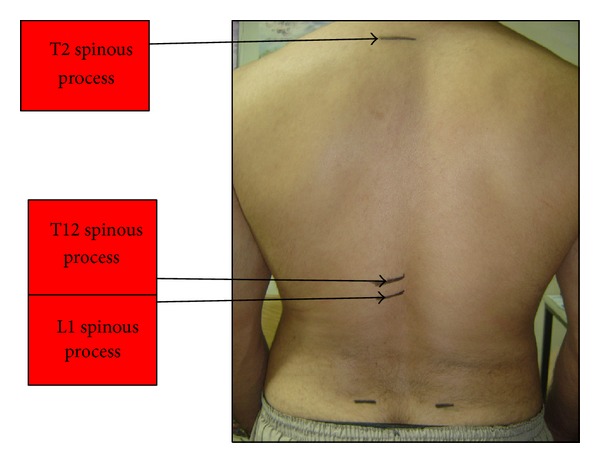
Locating T2, T12, and L1 spinous processes.

All participants signed an informed consent form. In order to measure the kyphosis, they were asked to take off dresses and shoes. For all students weight and height were measured. Using Adams test, participants were asked about history and sign of spinal surgery. For measuring the kyphosis, students were asked to stand comfortable and look forward in a way that body weight would be divided equally on both feet (arms beside the body). In this position, using surface anatomy and by palpation, spinous processes of 2nd and 12th thoracic vertebrae (T2 and T12) were determined and marked by a marker (Figure 1).

**Figure 2:**
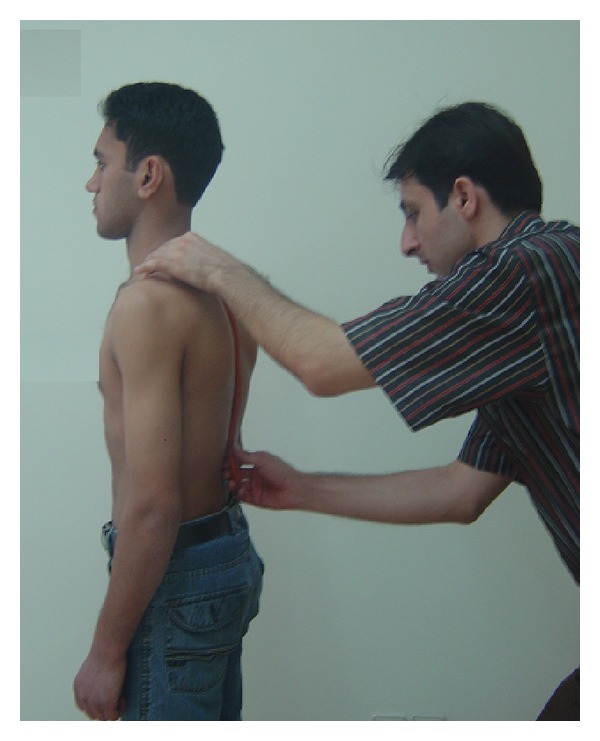
Placing flexible ruler on subjects backs for measuring its angle.

A flexible ruler was placed on thoracic curve covering the two marked points and pushed gently on back so there was no gap between the skin and the ruler (Figure 2). Keeping this position, a second coworker marked the adjacent points to T2 and T12 spinous processes on the ruler. Without making any change in the curve induced in the flexible ruler, it was put on a piece of paper. Using a pen, the curve was drawn and those two points were reflected on the paper. After removing the ruler a straight line was drawn connecting T2 and T12 points. The length of this line was measured and called “*l*”. Then distance between the deepest point of the curve and line “*l*” was measured as “*h*”. Using the equation below, magnitude of the angle of thoracic curve was measured [20]:

(1) $$\begin{array}{c} \theta =4\;\text{Arctang}\left(\frac{2h}{l}\right). \end{array}$$

Before starting the study, the reliability of the measurements was analysed using intraclass correlation coefficients (ICC). Forty-three subjects were tested 2 times with 5 minute interval. Data were analysed using SPSS version 11.5 software.

## 3. Results

Number of subjects in each age group and their height and weight characteristics are shown in Table 1. Using ANOVA statistic test (*P* < 0.001) it was found that mean height and weight of subjects in age groups were significantly different. By performing the measurement 2 times with 5 minute interval, intraclass correlation coefficients (ICC) were found high (86%); therefore a good reliability of this test was confirmed.  Table 2 shows mean kyphosis angle with 95% confidence interval in each age group (13–18). Mean thoracic kyphosis for each age group was 13 (34.41°), 14 (34.86°), 15 (35.79°), 16 (36.49°), 17 (35.84°), and 18 (35.55°).

When the whole population is considered, the average kyphosis was 35.49° SD 7.83, ranging from 12.05° to 68.84°. This study would indicate that 95% confidence interval of normal kyphosis was between 19.83° to 51.15°. Using ANOVA statistic test (*P* < 0.001) it was found that mean kyphosis of subjects in 13–18-year groups was significantly different too.

**Figure 3:**
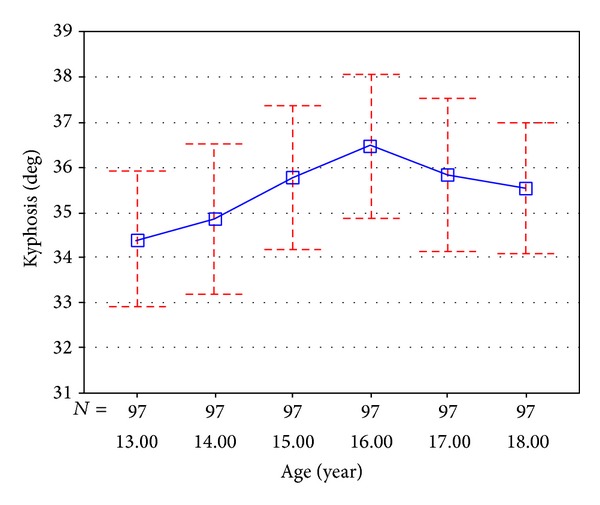
Thoracic kyphosis change in age groups (*n* = 582).

With increase in age from 13 to 16 years old, the magnitude of mean of kyphosis increased from 34.41° to 36.49° and then there was a mild decrease reaching to 35.5° by the age of 18 years old (Figure 3). The least thoracic angle was 12.05° observed in students who were 13 years old and the biggest was 68.84° in those who were 16 years old.

## 4. Discussion

The goal of this study was determination of thoracic kyphosis “normalcy” in male children and adolescents ranging from 13 to 18 years of age for regional population.

Most researchers believe that there is an increase in thoracic kyphosis in the age of 10 to 20 in both sexes. Mac-Thiong et al. [21] reported the tendency of kyphosis to increase linearly with age for children and adolescents. Our research confirms this increase from 13 to 18 too. Fon et al. [2] stated that the increase in the kyphotic curve with age is not unexpected, because of the associated changes in the soft tissues and mineral content of the bones with the progression of years. An association of progressive increase in spinal curvatures with gradual compression wedging of the vertebrae and its narrowing of the intervertebral discs has been described. Age-related increases in thoracic kyphosis can be attributed to overloading spinal soft tissue, particularly to the intervertebral disk with aging, during the growth period.

Fon et al. [2], Willner and Johnson [13], Voutsinas and MacEwen [15], Boseker et al. [18], Mac-Thiong et al. [21], Poussa et al. [22], and Cil et al. [23] in their articles have reported the least pronounced kyphosis at the age of 10–12 years, but, because we did not choose subjects in this range, the least kyphosis in our study was seen in the 13-year-old group.

The biggest magnitude of mean curve was seen in 16-year-old age group that may be due to pubertal growth. A positive correlation was seen between the velocity of growth and the range of the kyphosis in Willner and Johnson [13], Poussa et al. [22], and Cil et al. [23] studies. In the present study we cannot conclude this correlation because the puberties of subjects were not assessed.

Most researches have shown an age-related trend toward increases in thoracic kyphosis. However, some decreases were observed in isolated groups. For example, Willner and Johnson [13], Poussa et al. [22], and Giglio and Volpon [24] reported some decreases at 15, 14, and 15-16 years of age, respectively. Our subjects demonstrated a decrease in 17-18. The age for this decrease in each research was different which can be due to natural differences in subjects (race, lifestyle, age of puberty related to geographic region, etc.), or margin of errors demonstrated in using different measurement methods. Giglio considered these isolated changes to be a random occurrence.

Radiography (Cobb method) is the most commonly used method to assess sagittal spinal curves. Some studies using radiography including Takemitsu and Harada [25] in a study on 519 students reported 37° for mean thoracic kyphosis. Fon et al. [2] (measuring superior border of the upper end vertebra as well as along the inferior border of the lower end vertebra) found 25.11° for 10–19-year-old boys. Probst Proctor and Bleck [14] also measured the T5–12 area in 104 normal children. The mean value was 27° (range, 2°–33°). Vedantam et al. [26] measured the thoracic kyphosis from the upper end plate of T3 to the lower end plate of T12 in 88 boys and girls of 10–18 years of age. Mean angle was 38.00°. Boseker et al. [18] in a study on 121 subjects of 5–19 years of age stated the mean angle 33.00° with normal range (mean ± 2SD) of 20–50°. In Mac-Thiong et al. [21] study, values of 38.3° and 44.2° were reported for children younger than 10 years old and subjects of 10 years of age or older (both sexes), respectively. And Cil et al. [23] in a study of both male and female showed the average to be 45.8° for 10–12 and 53.3° for 13–15 years of age.

Although radiography is the most commonly used, this method is not ideal for systematic population studies because of its high cost and exposure of subjects to ionizing radiation. Some radiograph images are difficult to analyze, as it may be hard to precisely identify the beginning of the kyphotic curve because of the shoulder girdle and rib overlap. To overcome these limitations, thoracic curve was clinically evaluated by other instruments.

Willner and Johnson [13] studied the thoracic kyphosis in 1101 healthy children in consecutive age groups between 8 and 16 years of age using spinal pantograph. Their mean values for different ages were 13 (31.9°), 14 (37.1°), 15(35.6°), and 16 (37.4°). Using the same instrument, Poussa et al. [22] results for boys in 11, 12, 13, and 14 years of age were 27.8°, 28.0°, 30.9°, and 30.0°, respectively. Widhe [27] measured the thoracic curve using kyphometer on 46 males of 15-16 years and the mean value was 33.7°. And Giglio and Volpon [24] using spinal pantograph showed 33°, 38°, 35°, 32°, 36°, and 37° for thorasic kyphosis in 13, 14, 15, 16, 17, and 18 year old boys, respectively.

To investigate the comparative validity and the intra- and interrater reliability of thoracic kyphosis measurements using the flexicurve method, Teixeira and Carvalho [28] conducted a study in which the thoracic kyphosis was evaluated from sagittal radiography of the thoracic column using Cobb's method and by means of the flexicurve method. The intraclass correlation coefficient (ICC) between two measurements was 0.91.

Estimates of intertrial and interrate reliability of spine curvature measures acquired using the digital inclinometer and flexicurve ruler also were similar [29].

As different instruments with different methods are used in each study and because of subject's variation in different regions and races, there is a wide range of results for mean thoracic kyphosis. In nine mentioned studies on 10 to 20 years of age boys, it ranged from 25.11° to 53.3°. Our results in the present study (from 34.41° to 36.49°) are in agreement with them.

Generally, the values driven by radiographic method are obviously more than those taken by noninvasive methods. This may be due to difference in measuring techniques.

## 5. Conclusions

Using flexicurve ruler in measuring thoracic kyphosis which is ideal for systematic population studies, a norm was established for male students aged 13 to 18 years old which can be used as normal values for local and regional purposes. Our results are in agreement with previous reports.

## Figures and Tables

**Table 1:** Subject's characteristics (Mean ± SD).

Age groups (y)	Number (*n*)	Height (cm)	Weight (kg)
13	97	156.99 ± 8.76	48.02 ± 9.97
14	97	165.06 ± 7.42	53.24 ± 11.06
15	97	168.07 ± 7.44	56.32 ± 9.82
16	97	174.28 ± 6.22	64.68 ± 10.23
17	97	175.70 ± 5.88	67.14 ± 7.62
18	97	176.32 ± 6.19	69.46 ± 8.09

Sum	582		

**Table 2:** Mean and normal range of thoracic kyphosis in each age group.

Age group (year)	Normal range*	(Mean ± SD)
13	19.47 ± 49.34	34.41 ± 7.47
14	18.29 ± 51.44	34.86 ± 8.29
15	19.92 ± 51.65	35.79 ± 7.93
16	20.78 ± 52.20	36.49 ± 7.85
17	19.18 ± 52.50	35.84 ± 8.33
18	21.42 ± 49.69	35.55 ± 7.07

*Normal range in each population is mean ± 2SD.
